# One case of surgical treatment of coagulation factor XI deficiency complicated with esophageal cancer: a case report

**DOI:** 10.1186/s13019-024-02494-4

**Published:** 2024-02-02

**Authors:** Yang Tian, Zhaowei Zheng, Nannan Song, Jun Wang

**Affiliations:** Department of Cardiothoracic Surgery, Hangzhou Hospital of Traditional Chinese Medicine, Hangzhou, 310000 China

## Abstract

**Background:**

Coagulation factor XI deficiency is an autosomal recessive hereditary disease with a low incidence. It usually occurs after surgery or trauma; Esophageal cancer is a common malignant tumor of the digestive tract in China. But so far, surgery-based comprehensive treatment of esophageal cancer still dominates.

**Case presentation:**

We report a case of an Asian patient with XI factor deficiency and lower esophageal squamous cell carcinoma who was admitted to our hospital recently. After active preoperative preparation, the operation was successfully performed, and there was no obvious abnormal bleeding during and after the operation.

**Conclusions:**

Coagulation factor XI deficiency is a relatively rare disease, and patients with the disease will face a greater risk of bleeding during the perioperative period. The encouraging perioperative outcome enables us to have a deeper understanding of surgical treatment strategies for patients with Coagulation factor XI deficiency.

## Background

Coagulation factor XI deficiency is an autosomal recessive genetic disease with a low incidence rate, The prevalence of severe FXI deficiency is reported to be 1:10^6^ worldwide, and mild-to-moderate FXI deficiency is more common, with a prevalence of 1:10,000–50,000. The bleeding symptoms are milder than those of hemophilia A and hemophilia B. Spontaneous bleeding is rare [1, 2]. This article summarizes a recent case of coagulation factor XI deficiency with lower esophageal squamous cell carcinoma treated in our center, and the report is as follows.

## Case presentation

### Materials and methods

#### General situation

A 74-year-old male patient was admitted to the hospital with “progressive dysphagia discomfort for 4 months”. Previously healthy, no family history of unexplained bleeding or malignancy. Physical examination: The neck and supraclavicular lymph nodes were not palpable and enlarged, and the cardiopulmonary and abdominal examinations were unremarkable. Auxiliary examination: gastroscopy: Exophytic growth changes could be seen from the esophagus to the incisors at 30–33 cm, easy to bleed when touched. The pathological lumen was narrow, and esophageal cancer was considered first; biopsy pathology: poorly differentiated squamous cell carcinoma. Initial diagnosis: Esophageal squamous cell carcinoma.

#### Admission examination

Blood routine: white blood cell count (WBC) 4.77 × 10^9^/L, red blood cell count (RBC) 3.56 × 10^12^/L, hemoglobin (Hb) 107 g/L, platelet count (PLT) 112 × 10^9^/L. Coagulation function: prothrombin time (PT) 11.8S, international normalized ratio (PTINR) 1.02, partial thromboplastin time (APTT) 85.9S, fibrinogen (Fib) 3.54 g/L, thrombin time (TT) 17.1 S. Plasma coagulation factor determination: plasma coagulation factor II determination 99.7%, plasma coagulation factor V determination 98.2%, plasma coagulation factor VII determination 75.0%, plasma coagulation factor VIII determination 124.6%, plasma coagulation factor IX determination 87.1%, plasma coagulation factor X determination 68.8%, plasma coagulation factor XI was determined to be 0.7%.

**Fig. 1:**
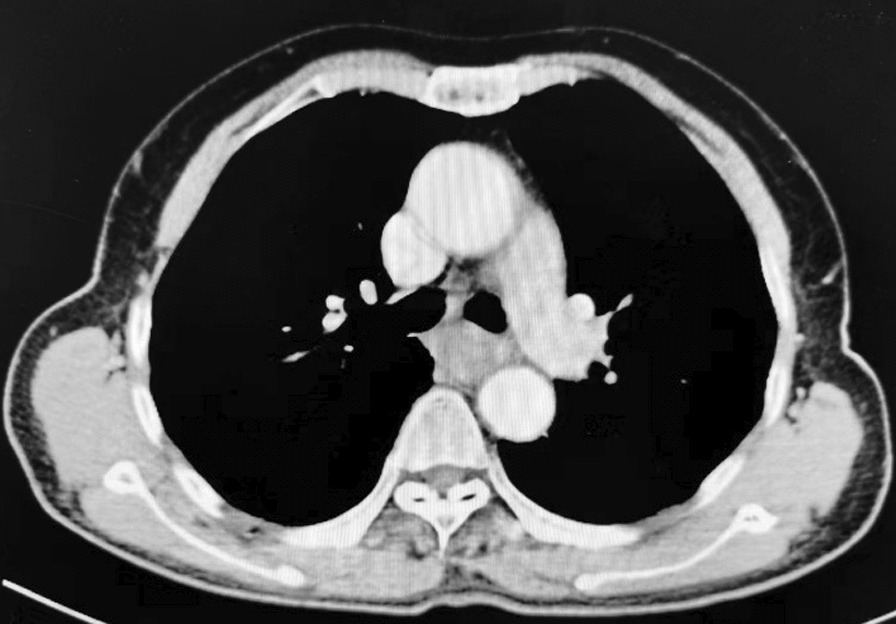
Enhanced CT of the chest

Enhanced CT of the chest: The wall of the middle and lower segments of the esophagus was thickened, and the thicker wall was about 1.7 cm. The contrast-enhanced scan showed mild to moderate enhancement, and local patchy low-enhancement areas were seen. The gap between the lesion and the fat of the thoracic aorta was not clear. Conclusion: the wall of the middle and lower esophagus is thickened, and there is no obvious enlarged lymph node in the mediastinum and esophageal cancer is considered (Fig. 1).

Upper Gastrointestinal Angiography: Consider CA in the middle and lower esophagus.

Abdominal enhanced CT, supraclavicular lymph node ultrasonography, cervical lymph node ultrasonography, and bronchoscopy showed no obvious signs of adjacent organ invasion or metastasis.

#### Multidisciplinary consultation

The coagulation function of the patient indicated that the APTT was significantly prolonged, and the plasma coagulation factor XI was significantly decreased (Normal range: 70–120%). The patient's medical history was inquired, and there was no history of gingival, oral mucosa, or other bleeding symptoms; multidisciplinary consultation was requested from the hematology department, blood transfusion department, and medical oncology department. F11 Exon detection, detection results: Exon 13 has a heterozygous mutation c.1556G > G/A and causes partial amino acid changes; Supplementary diagnosis: coagulation factor XI deficiency.

### Treatment

#### Preoperative replacement therapy

Fresh frozen plasma (FFP) was infused from 1 week before surgery to operation day, at a dose of 15 mL/kg/d. At the same time, the levels of APTT and FXI were detected daily, and the dosage of FFP was adjusted according to the test results. Normal level (≤ 37 s) (See Table 1 for the daily infusion volume of FFP and the levels of APTT and FXI).

**Table 1:**
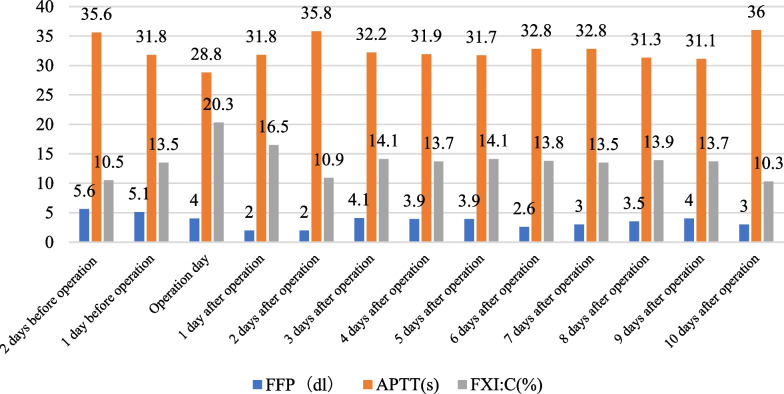
Perioperative FFP infusion volume and changes in APTT and FXI:C

#### Surgical treatment

The patient and his family refused neoadjuvant therapy and strongly requested surgery. After the maintenance of preoperative replacement therapy, a minimally invasive esophagectomy (MIE) was performed. The operation went smoothly. The intraoperative blood loss was about 200 ml, and no obvious abnormal bleeding was found.

#### Postoperative replacement therapy

From the day of operation to the 10th day after operation, fresh frozen plasma (FFP) was continuously infused. (Daily infusion volume of FFP, APTT, and FXI levels are shown in Table 1.

### Recent prognosis

The patient recovered smoothly after the operation, no signs of spontaneous bleeding were seen in the skin and mucous membranes of various parts, and no obvious abnormality was found in the properties of the pleural drainage fluid (Tables 2 and 3).

**Table 2:**
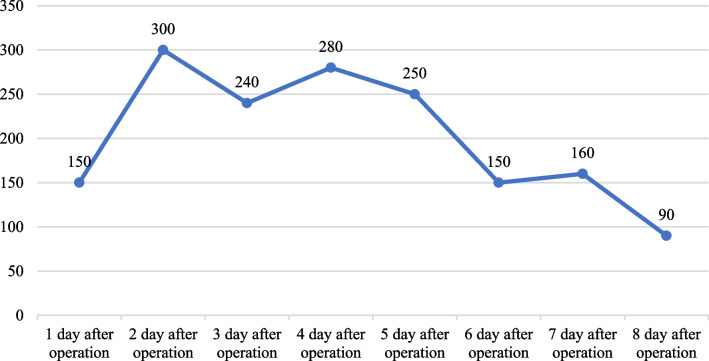
Postoperative thoracic drainage (mL)

**Table 3:**
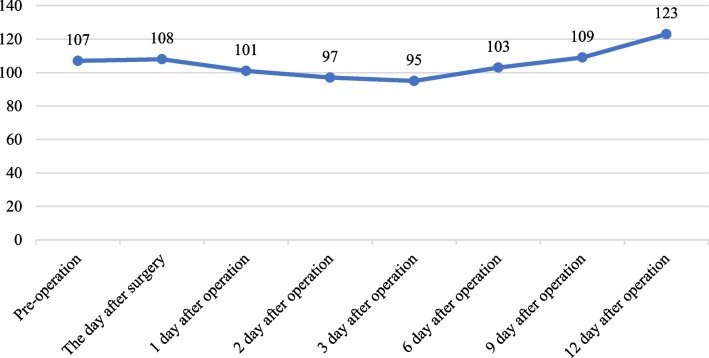
Perioperative hemoglobin level (g/L)

### Postoperative diagnosis

Postoperative diagnosis: mid-esophageal squamous cell carcinoma (pT3N1M0).

## Discussion

Coagulation factor XI is a serine protease synthesized by hepatocytes and megakaryocytes and exists in peripheral blood in the form of dimers in vivo. The main activator of FXI is thrombin, and activated FXI can activate coagulation factor IX to participate in the coagulation cascade reaction. At the same time, FXI can promote the activation of fibrinolysis inhibitors [3], which stabilizes the formed clots. FXI deficiency (also known as hemophilia C) is an autosomal recessive genetic disease caused by mutations in the FXI gene. There was no significant correlation between the degree of bleeding and the degree of FXI deficiency; it was mostly found due to surgery, trauma, massive bleeding in the nasal and oral mucosa, or marked prolongation of APTT on examination [4, 5]. Up to now, a total of at least 40 genetic mutations related to the genetic coagulation factor XI have been reported. Among them, 22 are missense mutations, 7 are nonsense mutations, 5 are insertions or deletions of bases or nucleic acid fragments, and 6 are splice site abnormalities leading to abnormal splicing of mRNA. There is little literature on the perioperative treatment and management of coagulation factor XI deficiency complicated with esophageal cancer. Combined with the perioperative treatment of this patient, we have a deeper understanding of the patient with coagulation factor XI deficiency combined with surgery.

Clinical symptoms and diagnosis of the disease: The coagulation function of patients with coagulation factor XI is generally characterized by normal prothrombin time (PT), normal thrombin time (TT), and markedly prolonged partial thromboplastin time (APTT). The determination of FXI activity (FXI: C) has diagnostic significance. The FXI activity level is lower than the normal value (50%-150%), which can be diagnosed [6]; this disease is mainly related to bleeding disorders with normal prothrombin time and prolonged partial thromboplastin time (APTT). Identification, Biggs thromboplastin generation test can be differentiated from hemophilia A and B, and laboratory tests of lupus anticoagulant substances and FXI autoantibody tests can differentiate systemic lupus erythematosus.

Perioperative replacement therapy: Infusion of fresh frozen plasma is currently the main perioperative treatment for coagulation factor XI deficiency patients. The half-life of FXI in FFP is about 45 h, the infusion dose of FFP is usually (7–20)mL/kg, the therapeutic dose is usually 15 mL/kg/d, the APTT in the perioperative period should be controlled for less than 50 s, and FXI: C greater than 50% However, considering that the content of FXI varies greatly in different batches of FFP, FFP infusion also needs to dynamically monitor APTT and FXI levels, and adjust the infusion dose in time according to the results [7]; The therapy program has been successfully reported in lung cancer surgery in the field of thoracic surgery[8], but there is no literature report on esophageal cancer surgery. In addition, for patients with severe bleeding, FXI concentrates and recombinant rFVIIa have also been used clinically to provide options for patients with coagulation factor XI deficiency [9].

Our successfully applied FFP replacement therapy to perform surgery on a patient with coagulation factor XI deficiency and lower esophageal squamous cell carcinoma. The patient had no signs of spontaneous bleeding before. After admission, it was confirmed to be coagulation factor XI according to coagulation function, plasma coagulation factor levels, and genetic testing. deficiency. We infuse the patients with FFP according to the infusion dose (15) mL/kg before surgery. After the replacement therapy, we focus on the APTT level, control the perioperative APTT to a normal level (≤ 37 s), and adjust the FFP infusion according to the APTT level. The dosage was continued until the patient's condition stabilized on the 10th postoperative day, and the infusion of FFP was stopped. In this case, no other antifibrinolytic drugs such as tranexamic acid were used in the perioperative period except FFP. There were no obvious bleeding complications during and after the operation and no obvious signs of bleeding in the thoracic drainage tube and the indwelling gastric tube. Combined with current literature reports, replacement therapy for coagulation factor deficiency has been applied in lung tumors, craniocerebral surgery, femoral head replacement, breast cancer, and other surgeries and achieved ideal results [10, 11], However, due to individual differences, patients have different degrees of coagulation factor deficiency and different treatment responses after FFP infusion. It is still necessary to carry out individualized treatment according to the patient's condition to ensure that the APTT level, FXI: C, and other indicators after replacement therapy meet the surgical requirements and ensure the safety of surgery.

## Conclusion

Patients with coagulation factor XI deficiency complicated with surgical diseases are relatively rare, and identifying and diagnosing coagulation factor XI deficiency is very important. Monitoring APTT and FXI: C and other indicators according to individual differences of patients can effectively prevent and treat perioperative bleeding, and FFP-based alternative therapy provides a guarantee for surgery. However, patients with coagulation factor XI deficiency combined with surgical treatment are rare, and the related perioperative replacement therapy still needs to be further explored.

## Data Availability

Data is available on request from the authors.
